# Proteomic Analysis of Methanonatronarchaeum thermophilum AMET1, a Representative of a Putative New Class of Euryarchaeota, “Methanonatronarchaeia”

**DOI:** 10.3390/genes9020028

**Published:** 2018-01-23

**Authors:** Manuel Ferrer, Dimitry Y. Sorokin, Yuri I. Wolf, Sergio Ciordia, María C. Mena, Rafael Bargiela, Eugene V. Koonin, Kira S. Makarova

**Affiliations:** 1 Institute of Catalysis, CSIC, 28049 Madrid, Spain; mferrer@icp.csic.es (M.F.); rafaelbb@icp.csic.es (R.B.); 2 Winogradsky Institute of Microbiology, Research Centre for Biotechnology, Russian Academy of Sciences, Prospect 60-let Octyabrya 7/2, 117312 Moscow, Russia; D.Sorokin@tudelft.nl; 3 Department of Biotechnology, Delft University of Technology, van der Maasweg 9, 2629 HZ Delft, The Netherlands; 4 National Center for Biotechnology Information, National Library of Medicine, National Institutes of Health, Bethesda, MD 20894, USA; wolf@ncbi.nlm.nih.gov (Y.I.W.); koonin@ncbi.nlm.nih.gov (E.V.K.); 5 Proteomics Facility, Centro Nacional de Biotecnología, CSIC, 28049 Madrid, Spain; sciordia@cnb.csic.es (S.C.); mcmena@cnb.csic.es (M.C.M.)

**Keywords:** proteomics, methanogenesis, halophiles, Methanonatronarchaeia, genomics

## Abstract

The recently discovered Methanonatronarchaeia are extremely halophilic and moderately thermophilic methyl-reducing methanogens representing a novel class-level lineage in the phylum Euryarchaeota related to the class Halobacteria. Here we present a detailed analysis of 1D-nano liquid chromatography–electrospray ionization tandem mass spectrometry data obtained for “Methanonatronarchaeum thermophilum” AMET1 grown in different physiological conditions, including variation of the growth temperature and substrates. Analysis of these data allows us to refine the current understanding of the key biosynthetic pathways of this triple extremophilic methanogenic euryarchaeon and identify proteins that are likely to be involved in its response to growth condition changes.

## 1. Introduction

Methanogenesis is a major biogeochemical process that is indispensable for the global carbon cycle and climate maintenance. Currently, only archaea have been conclusively shown to produce methane at anaerobic conditions in nature [1,2]. Methanogenic archaea belong to several distinct lineages. The best characterized ones are members of the phylum Euryarchaeota and include the classes Methanomicrobia, Methanobacteria, Methanopyri, Methanococci, the recently discovered candidate classes “Methanonatronarchaeia” and “Methanofastidiosa” and the order Methanomassiliicoccales within the class Thermoplasmata. Several putative methyl-reducing methanogens belong to the candidate phyla “Bathyarchaeota” and “Verstaraetearchaeota” that are classified within the Thaumarchaeota-Aigarchaeota-Crenarchaeota-Korarchaeota (TACK) superphylum [2,3]. Several methanogens that apparently represent additional, diverse archaeal groups so far have been identified only in metagenomic samples and the respective genomes are expected to become available in the near future [2]. Reconstruction of the gene gain and loss in the evolution of archaea suggests that the last archaeal common ancestor (LACA) already encoded the enzymatic machinery for methanogenesis that was lost independently on several occasions during archaeal evolution [3,4,5]. 

“Methanonatronarchaeia” are a recently discovered candidate euryarchaeal class with a unique phenotype and a distinct architecture of the methanogenic pathways [3]. These organisms were isolated from hypersaline salt and soda lakes located in south-eastern Siberia where high activity of classical methylotrophic methanogenesis has been detected at ambient temperature, while at elevated temperature it was outcompeted by methyl-reducing “Methanonatroarchaeia”. Currently, this class is represented by two distinct genera that include, respectively, 10 pure cultures of alkaliphilic *Methanonatronarchaeum thermophilum* from soda lakes, some of which can be grown in a defined medium and “*Candidatus* Methanohalarchaeum thermophilum” from salt lakes, which so far is represented by three highly enriched mixed cultures with some other haloarchaea [3]. Furthermore, 16S rRNA sequences classified as SA1 group [6,7,8] that are forming coherent cluster with “Methanonatronarchaeia,” were identified at the brine-seawater interface of the Shaban Deep in the Red Sea and some other hypersaline sites, suggesting that organisms from this class could be widespread in the hypersaline environments [3]. 

*M. thermophilum* AMET1 (type strain, with AMET is standing for Alkaliphilic Methylotyrophic Thermophile) is an extremely haloalkaliphilic and moderately thermophilic, methyl-reducing methanogen with very small motile coccoid cells [3]. It optimally grows at 48–50 °C, 4 M total Na^+^, pH 9.5 with methanol (MeOH) and formate as the electron acceptor-electron donor, respectively. Yeast extract or acetate (less actively) can be utilized as carbon source. *M. thermophilum* AMET1 is the first methanogen with salt-in osmotic strategy which is also corroborated by its phylogenetic relation to haloarchaea [3]. AMET1 is a methyl-reducing methanogen utilizing various C1-methylated compounds as the e-acceptor and formate or H_2_ as the e-donor. Almost complete genomic sequences have been obtained for both *M. thermophilum* and “*Candidatus* Methanohalarchaeum thermophilum” HMET1 (hereinafter HMET1, for Halophilic Methylotrophic Thermophile) and comparative genomic analysis has allowed the reconstruction of the key metabolic networks and cellular processes in these organisms [3]. This analysis revealed that the two organisms share a distinct set of metabolic genes compared to other known methanogens [3]. For both organisms, proteomic data for optimal growth conditions have been reported but have not been analysed in detail [3]. Here we report the analysis of the two proteomes of “Methanonatronarchaeia” and compare them with new proteomic data obtained for *M. thermophilum* AMET1 growing under five different conditions including variation of the growth temperature and key components required for methyl reduction pathways.

## 2. Methods

### 2.1. Cultivation Conditions and Proteomics

Strain AMET1 was cultivated in 150 mL serum bottles with 120 mL liquid medium containing 4 M total Na^+^ (50% as NaCl and 50% as sodium carbonates) strongly buffered at pH 9.5. After sterilization, the base medium was supplemented with 1 mL/L of acidic and alkaline (W/Se) trace metal solutions, 1 mL/L of vitamin mix, 4 mM NH_4_Cl, 100 mg/L yeast extract, 0.1 mM filter-sterilized CoM and either with 0.2 mM colloidal FeS (hydrotroillite) or 1 cm^3^ of heat sterilized anaerobic sediments from Siberian hypersaline soda lakes. The medium was made anoxic with 5 cycles of argon flushing-evacuation and finally reduced by the addition of 1 mM Na_2_S and 20 μL/100 mL of 10% dithionite in 1 M NaHCO_3_. H_2_ was added on the top of argon atmosphere at 0.5 bar overpressure, formate and methanol—at 50 mM and trimethylamine—at 10 mM. In case of the latter, ammonium was omitted from the basic medium. The incubation temperature varied from 34 to 55 °C. The culture progress was monitored by analysing methane formation in the gas phase by the gas chromatography as described previously [9,10].

Proteomic analyses were conducted using the soda lake pure culture *M. thermophilum* AMET1 for the following conditions: “base” or optimal conditions −48 °C, 4 M total Na^+^, pH 9.5, MeOH as an electron acceptor and formate as an electron donor and sterilized sediments as growth factor; 34 °C, as the minimal possible growth temperature for this strain “34 °C”, otherwise optimal conditions; 55 °C, as the maximum possible growth temperature for this strain “55 °C”, otherwise optimal conditions; “TMA”—trimethylamine as an alternative electron acceptor (with much slower growth in comparison to methanol), otherwise optimal conditions; “FeS”—FeS as a growth factor (with slower and poorer growth in comparison to sterilized sediments), otherwise optimal conditions; “H_2_”—H_2_ as an electron donor (poorly soluble at saturated salt concentration and with slower growth in comparison to soluble formate), otherwise optimal conditions. Cultivation was performed in all cases as described above. 

Given the low growth rates of the pure culture of AMET1 under the different conditions tested and the highly resource-consuming nature of the proteomic analyses (more than 20 μg total protein) we decided to conduct shotgun proteomic analyses by combining cells from biological triplicates and further processing them as a single sample. We sacrificed the differences between replicates, which have been found to be minimal in similar previous studies, to investigate differences among cells from cultures obtained under different conditions. More in details, cell pellets obtained from three parallel cultures for each condition (except TMA with one replicate culture) were pooled and the resulting pellet dissolved in lysis buffer (8 M urea, 2 M thiourea, 5% 3-[(3-cholamidopropyl)dimethylammonio]-1-propanesulfonate (CHAPS), 5 mM Tris (2-carboxyethyl)phosphine (TCEP)-HCl and a protease inhibitors cocktail). Homogenization of the cells was achieved by ultra-sonication for 5 min on ultrasonic bath. After homogenization, the lysed cells were centrifuged at 20,000× *g* for 10 min at 4 °C and the supernatant containing the solubilized proteins was concentrated and used for liquid chromatography–tandem mass spectrometry (LC-MS/MS) experiment. Detailed descriptions of all methodological procedures used in this study for protein concentration, 1D-nano liquid chromatography–electrospray ionization tandem mass spectrometry, protein identification and confidence threshold based on the one-peptide rule [9] and data acquisition of the Exponentially Modified Protein Abundance Index (emPAI), are given in a previous work [3]. The emPAI was used as a relative quantitation score of the proteins in a complex mixture based on protein coverage by the peptide matches in a database search result [11].

### 2.2. Proteomic Data Normalization and Analysis

Normalized emPAI values (nemPAI, see *Proteomic data normalization and analysis* section in Methods) were obtained from the emPAI values by dividing each individual value by the sum of all emPAI values in a given experiment [12]; then multiplied by the number of proteins in *M. thermophilum* AMET1 distinct by their peptide composition (1529) to make the scale commensurate with the average protein abundance. Decimal logarithms of nemPAI values with all originally zero values replaced by artificial low values of 0.001 (the lowest observed non-zero abundance being 0.006) were used in most subsequent analyses (referred to as lPAI values).

When coarse-grained data was required for the correlation analysis, the following procedure was applied to each protein-specific vector of 6 lPAI values: the minimum lPAI value was subtracted from each data point, the result was divided by a threshold value (0.5 decimal log units, see Results for details) and rounded down to the nearest integer. This produced a stepwise scaling of the data in threshold value units.

Principal Component Analysis (PCA) was performed on the lPAI values using the prcomp function of R package with rescaling of the original data columns [13].

Normal equivalent of the distribution [14] of lPAI values for proteins with non-zero measured abundance was calculated as follows: the mean of the equivalent normal distribution *μ_e_* was set to the median lPAI value. The equivalent standard deviation *σ_e_* was calculated as 0.74 of the interquartile distance (as the interquartile distance of the normal distribution is 1.35 standard deviation units). Therefore, the z-score for the protein abundance can be calculated as *z* = (*x*−*μ_e_*)/*σ_e_* and the Bonferroni-corrected e-value as the standard normal distribution p-value of *z* multiplied by the number of distinct proteins (1529). Proteins with *z* >0 and e-values below 1 were considered super-abundant in the sense that their abundances exceeded the expectation of the abundance range under the assumption of the log-normal distribution.

### 2.3. Sequence Analysis 

*M. thermophilum* AMET1 proteins were assigned to most recent archaeal Clusters of Orthologous Groups, arCOGs in the course of the previous work [3]. The PSI-BLAST program was used to search protein sequences against non-redundant database at NCBI (National Center for Biotechnology Information) [15]. Protein sequences for selected families were aligned using MUSCLE [16]. Protein secondary structure was predicted using Jpred 4.0 [17]. Transmembrane helices in proteins were predicted using TMHMM 2.0 [18].

## 3. Results

### 3.1. Estimation of Significant Changes in Protein Abundance

To determine if a change in a protein abundance is likely to be biologically significant, it is essential to assess the level of non-biological noise in the data. Although this level is not directly measurable (due to the lack of precise reference data), the following approach allows an approximate estimation. Ribosomal proteins, specifically those 25 that are encoded within the ribosomal superoperon (AMET1_0469-AMET1_0497 on MRZU01000003.1), are present in the ribosome in equal stoichiometric ratios, so their relative abundances should be largely independent of the physiological conditions. Thus, variations of the measured relative abundances of these ribosomal proteins under different experimental conditions should be largely attributed to non-biological noise.

This analysis shows that the measurements have the root-mean-square deviation from the expectation by a factor of 1.8 (0.25 decimal log units). The threshold of 0.5 decimal log units (a factor of 3.16) renders 97% of the deviations insignificant. Therefore, in the further analysis, we called changes in a protein abundance as significant if they differed from the baseline measurements by a factor of 10^0.5^ = 3.16 or greater. 

### 3.2. Methanonatronarchaeum thermophilum AMET1 Protein Abundances under Optimal Growth Conditions

**Figure 1:**
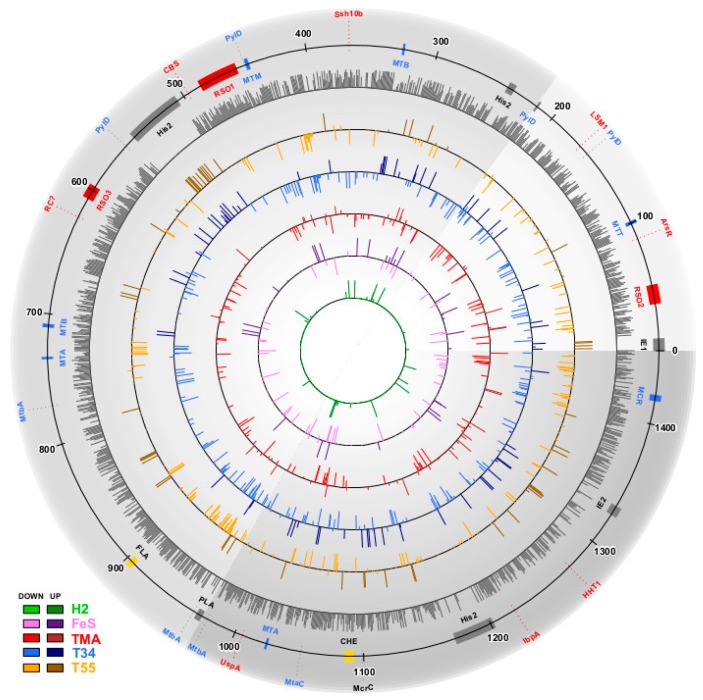
Schematic map of protein expression in different conditions along the *Methanonatronarchaeum thermophilum* AMET1 genomic contigs. The grey transparent sectors show *M. thermophilum* AMET1 genomic contigs as follows: AMET1_2–light grey, AMET1_3–medium grey, AMET1_4–dark dray (minor contigs are not shown). Dark grey circle shows base (optimal) conditions relative protein abundance level. The inner cycles represent up- and down regulated genes in different experimental conditions according to the colour code shown on the left bottom corner of the Figure. Genes and genes clusters described in the paper shown by shapes and lines as follows: Red—the most abundant genes; Red—RSO1,2,3—three ribosomal; Blue—methanogenesis related genes; Blue—methanogenesis operons: MTT-trimethylamine-corrinoid methyltransferase, MTB-dimethylamine-corrinoid methyltransferase superoperons; MTM-methylamine-corrinoid methyltransferase, MTA-methanol-corrinoid methyltransferase; MCR-methyl coenzyme M reductase; Yellow—chemotaxis operon (CHE) and archaellum (FLA). Black—mobile elements: His2—His2 virus related loci, PLA-putative plasmid, IE1 and IE2—integrated elements.

We identified 1026 (67%) of the 1529 predicted protein-coding genes of *M. thermophilum* AMET1 being expressed under the optimal growth conditions (Table S1). Most of the predicted proteins that have not been detected in the proteomic experiments belong to “silent” gene islands and the largest islands correspond to predicted integrated elements (Figure 1). Three other such regions are occupied by proviruses related to His2-like spindle-shaped haloviruses: partial AMET1_0244-AMET1_0250 and two apparently complete proviruses AMET1_0519-AMET1_0565, AMET1_1204-AMET1_1235, with integrases AMET1_0519 and AMET1_1204 respectively and His2-like major capsid proteins AMET1_0248, AMET1_0558 and AMET1_1226. These proviruses, however, could be replication-defective, because they do not appear to encode a family B DNA polymerase that is characteristic of His2 viruses [19]. Another island corresponds to a putative plasmid AMET1_0986-AMET1_0992, with two genes responsible for plasmid replication (AMET1_0989 and AMET1_0990). The silent islands also include uncharacterized integrated elements AMET1_0003-AMET1_0013 and AMET1_1363-AMET1_1373, with integrase AMET1_0013 and gene shared between the two islands (AMET1_0008 and AMET1_1363), suggesting that the elements might be related. The mechanism of silencing of such large DNA regions remains to be elucidated. Several transposable elements present in the genome are not expressed either. 

The distribution of non-zero nemPAI values is generally bell-shaped in logarithmic scale (Figure S1A). About 2% proteins have values of 9.0 or greater with 8 of these representing the super-abundant heavy tail of the distribution (Table 1). As expected, many of these proteins perform house-keeping functions, such as ribosomal proteins, chromatin, or RNA–binding proteins and chaperons. The archaeal DNA–binding protein Alba (AMET1_0379) shows the highest abundance value, 161 (Table 1). Alba is thought to play a key role in 3D chromosome organization and gene expression [20]. Another chromatin associated protein, histone, is also very abundant with nemPAI value 22. Notably, Alba is not particularly abundant in neutraphilic HMET1 compared with the histone, with nemPAI values 7 and 156 respectively [3]. Thus, it appears that chromosome organization is significantly different in these two organisms. Another group of highly abundant proteins is related to methyl coenzyme M reductase, a key multisubunit protein complex that is involved in the last enzymatic step of methanogenesis and is considered to be a hallmark of methanogens [21,22] (Table 1). This observation is consistent with extremely high abundances of these subunits in HMET1 and other methanogens for which proteomic data are available [3,23,24,25].

**Figure 2:**
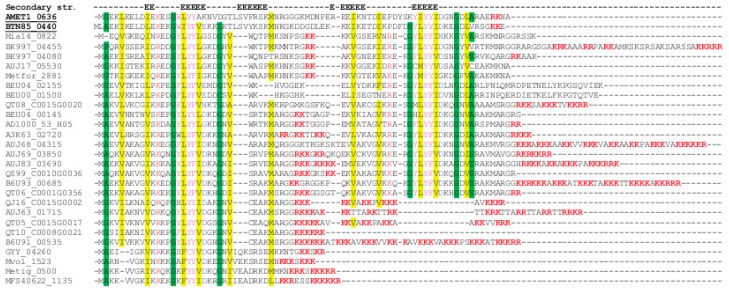
Multiple alignment of the predicted novel RNA chaperones. Multiple alignment is shown for selected archaeal representatives of the putative RNA chaperone family (see the complete list of homologs in the Table S2). The sequences are denoted by their locus tag numbers. Two underlined locus tag numbers correspond to *M. thermophilum* AMET1 and HMET1. Secondary structure elements are shown above the alignment, with “E” indicating positions predicted to be in a beta strand. Amino acids in the conserved (80%) positions are coloured according to their physico-chemical properties as follows: yellow background indicates aliphatic residues (I,L,M,V), green background indicates small residues (A,G,P,S), red letters indicate positively charged residues (K,R), blue—indicate negatively charged residues (D,E,N,Q), magenta-aromatic residues (F,Y,W). Patches of positively charged residues are highlighted in bold red font.

Finally, four abundantly expressed proteins are either assigned only a general function prediction or remain uncharacterized (Table 1). The first one is a transcriptional regulator containing a predicted winged helix-turn-helix domain (AMET1_0092, arCOG01060). In arCOGs, this subfamily is present in most of the methanogens but only in a few members of Halobacteria, which might point to involvement of this protein in regulation of methanogenesis pathways. Two other uncharacterized proteins belong to the CBS domain family (Table 1). These proteins are thought to be energy sensors that are involved in modulating various cellular metabolic processes [26]. There are numerous CBS proteins in *M. thermophilum* AMET1 including the cluster AMET1_0341-AMET1_0345 and most of these proteins are also highly abundant, suggesting important roles in metabolism regulation (Table S1). AMET1_0636 protein is not similar to protein family with known function. It has the largest nemPAI value (39.4) among uncharacterized proteins, with AMET1_1159 as a distant second, with nemPAI value of 8.0. Moreover, this protein has high nemPAI values under all conditions tested in this work (Table S1). Sequence analysis shows that homologs of this small protein (~40–90 amino acids (aa)) are present in many methanogens and other, mostly uncultivated archaea and bacteria (Table S2). Multiple alignment of selected proteins of this family is shown in Figure 2. Apparently, the core of the protein includes ~40 aa and, according to the secondary structure prediction, is an all-beta strand domain (Figure 2). AMET1_1159, like the majority of its homologs, consists of two duplicated domains whereas several archaea have only one domain which accounts for the variation in the protein length. In many prokaryotic species, these proteins contain variable positively charged amino acid patches (Figure 2). Such patches are typical of RNA chaperones [27]. The high abundance of AMET1_1159 suggests that it plays an important role in house-keeping cellular functions which is compatible with the predicted RNA chaperone function.

Next, we compared the abundance levels of different functional classes of proteins under the optimal conditions (Figure S1B). This comparison showed that the differences between functional classes are not substantial or statistically significant, with only proteins involved in translation showing an overall elevated abundance level.

Previously, we have found that the majority of proteins that are predicted to be involved in key metabolic pathways are produced in both *M. thermophilum* AMET1 and HMET1 as indicated by LC-MS/MS data analysis [3]. These include methyl-reduction pathway of methanogenesis, membrane respiratory chain and energy-converting membrane complexes, in particular, cytochromes, acetate incorporation pathway, CO_2_ fixation pathway through archaeal RUBISCO and a few other carboxylases, both glycolysis and gluconeogenesis pathway and others (Table S1). Most proteins involved in the biosynthetic pathways for all nucleotides, amino acids (including pyrrolysine), cofactors and lipids were also detected (Table S1). The lipid biosynthesis enzymes, however, as well as many transporters, are not well recovered, presumably, due to the known limitations of the protein preparation methods in dealing with proteins that are tightly associated with the membrane [28]. Only a few proteins that are also detected but were not included in previous reconstruction of the aforementioned pathways are functionally characterized and allow us to add a few more details to the *M. thermophilum* AMET1 metabolic map. In addition to the Suf system of Fe-S cluster formation, *M. thermophilum* AMET1 expresses the Isc system that is much more abundant than Suf. Several Fe-S clusters containing redox proteins that are common in methanogens but rare in *Halobacteria* are also abundant, namely NorV-like flavorubredoxin (AMET1_0320) and rubrerythrin (AMET1_1450 and AMET1_0761). Like in most archaea, synthesis of aromatic amino acids in *M. thermophilum* AMET1 proceeds via an alternative pathway in which the two first steps of 3-dehydroquinate biosynthesis are catalysed by 2-amino-3,7-dideoxy-D-threo-hept-6-ulosonic acid synthase (AMET1_1249) and 3-dehydroquinate synthase and (AMET1_1248) [29]. Both are detected in our proteomic data (Table S1).

Several proteins detected by proteomic analysis could not be confidently connected to the predicted metabolic network of AMET1. These include proteins comprising part of the hydrogenotrophic methanogenesis pathway, namely, coenzyme F420-dependent N(5),N(10)-methylene tetrahydromethanopterin reductase (Mer), coenzyme F420-dependent N(5),N(10)-methenyltetrahydromethanopterin dehydrogenase (Mtd), methenyltetrahydromethanopterin cyclohydrolase (Mch) and formylmethanofuran-tetrahydromethanopterin formyltransferase (Ftr). As discussed previously, the reactions catalysed by these enzymes could not be connected to the rest of the methyl-reducing pathway because of the absence of genes for Mtr and Fdw complex subunits [3]. Nevertheless, all these proteins were detected by proteomic analysis (Table S1), confirming their functionality and suggesting that an unknown link exists between these reactions and the rest of the metabolic flow and remains to be identified in further experiments. 

The UbiX-UbiD decarboxylase system is common among archaea, especially in methanogens. It has a considerable variety in terms of substrate specificity and physiological role [30]. Originally, it has been characterized as prenyltransferase required for bacterial ubiquinone biosynthesis but this pathway is absent in *M. thermophilum* AMET1 and most other methanogens, so the function of these proteins remains unknown [31]. We noticed, however, that in some archaea, including AMET1, *ubiX* and/or *ubiD* genes belong to the same gene neighbourhood with aconitase *acoX*, which is implicated in tricarboxylic acid cycle (TCA) [32] (Figure S2). The TCA reactions predicted from the gene content are disconnected in *M. thermophilum* AMET1 [3]. Thus, this organism might possess a yet unknown modification of the TCA that could require the carboxylase activity of UbiX-UbiD. Alternatively, both AcoX and UbiX-UbiD carboxylase enzymes might comprise a new pathway not related to TCA. The intriguing possibility is that they might be involved in methanophenazine biosynthesis pathway, a specific methanogenic variant of respiratory quinone derivatives that are present in many archaea and but for most of them the biosynthesis pathway is not yet known [33,34].

### 3.3. The Structure of the Protein Abundance Space in Different Conditions

**Figure 3:**
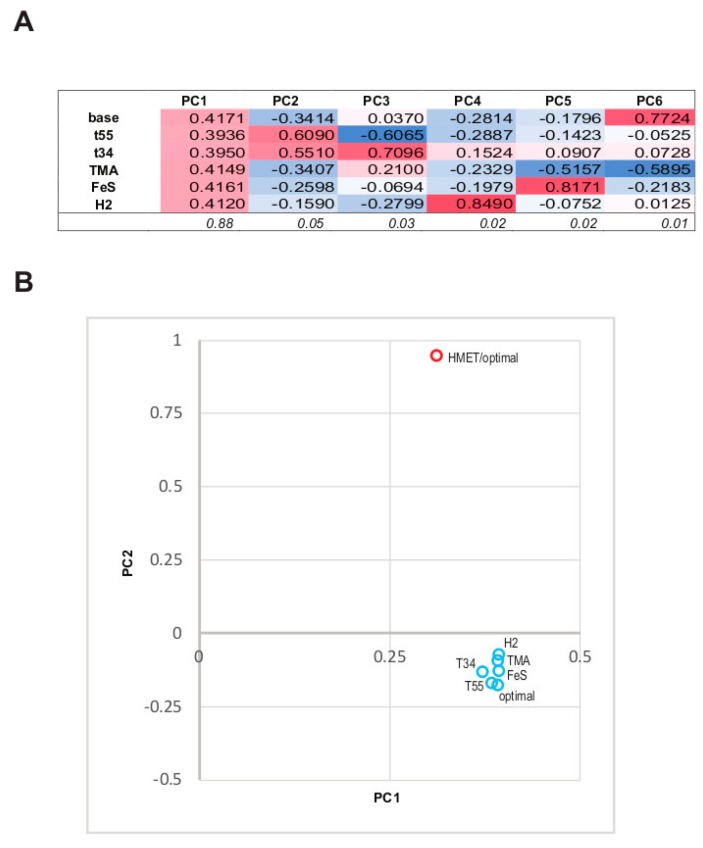
Statistical analysis of the protein abundance space. (**A**) Principal Component (PC1-PC6) loadings. The colour scale highlights the sing and the magnitude of the contributions of the individual abundance profiles to the Principal Components. (**B**) Comparison of the abundance of “*Candidatus* Methanohalarchaeum thermophilum” HMET1 proteins under optimal conditions with all six conditions tested for *M. thermophilum* AMET1. Data points represent the six *M. thermophilum* AMET1 profiles and the HMET1 profile, plotted against the first two Principal Components.

In order to determine the impact of different growth conditions (optimal, sub- and supraoptimal temperature, trimethylamin as an electron acceptor, FeS as growth factor, H_2_ as electron donor) on the abundance of 1157 unique quality-filtered proteins obtained from the 6 sample groups, we applied PCA to the proteomic data (Figure 3A). The first Principal Component (PC1) encompasses high positive contributions of abundances from all experimental conditions and these contributions are comparable in magnitude (Figure 3A). PC1 captures most of the original data variance, 88%. This indicates that, overall, the abundances in all experimental conditions are strongly and positively correlated and that the condition-independent component of the abundance is the dominant trend in the data. PC2 and PC3 are largely composed of the contributions of the two suboptimal growth temperature conditions; these contributions have the same signs in PC2 and opposite signs in PC3, together accounting for further 7.5% of the data variance. The remaining components are largely composed of individual contributions from experiments with changed trophic conditions that exert uncorrelated effects on the structure of the data space.

The observed relationship between the experimental conditions allows for a straightforward interpretation of the data. Because the abundances of most proteins are highly correlated between the experiments whereas different conditions produce largely orthogonal (uncorrelated) effects on different genes (except for the suboptimal temperature conditions that seem to exert some common influence), the optimal conditions data appear to be a natural choice for the baseline abundance values, whereas the protein abundances in the 5 experiments with altered conditions demonstrate largely uncorrelated deviations from this baseline.

We also compared the 6 abundance profiles for *M. thermophilum* AMET1 with the single profile for HMET1 (at optimal growth conditions). As shown in Figure 3B, the HMET1 profile is sufficiently similar to all *M. thermophilum* AMET1 profiles to make the common signal dominant in the principal components space. However, all *M. thermophilum* AMET1 data points group closely together compared to the distance from the HMET1 profile, suggesting that evolutionary divergence affects protein expression to a much greater extent than the changes in growth conditions covered by our experiments.

### 3.4. Supra- and Suboptimal Temperature Effect on Protein Abundance

Both cold and heat shock responses typically affect expression of hundreds of genes, most of which are organism-specific [35,36,37]. In archaea, these responses have been studied in several model organisms, mostly using transcriptomics. A major common trend that is shared not only by archaea but also by bacteria and eukaryotes, involves the heat-induced over-expression of a distinct set of highly conserved heat shock proteins, most of which are molecular chaperones [38]. Compared to the heat shock response, the response to cold is apparently more species-specific [36,39].

**Figure 4:**
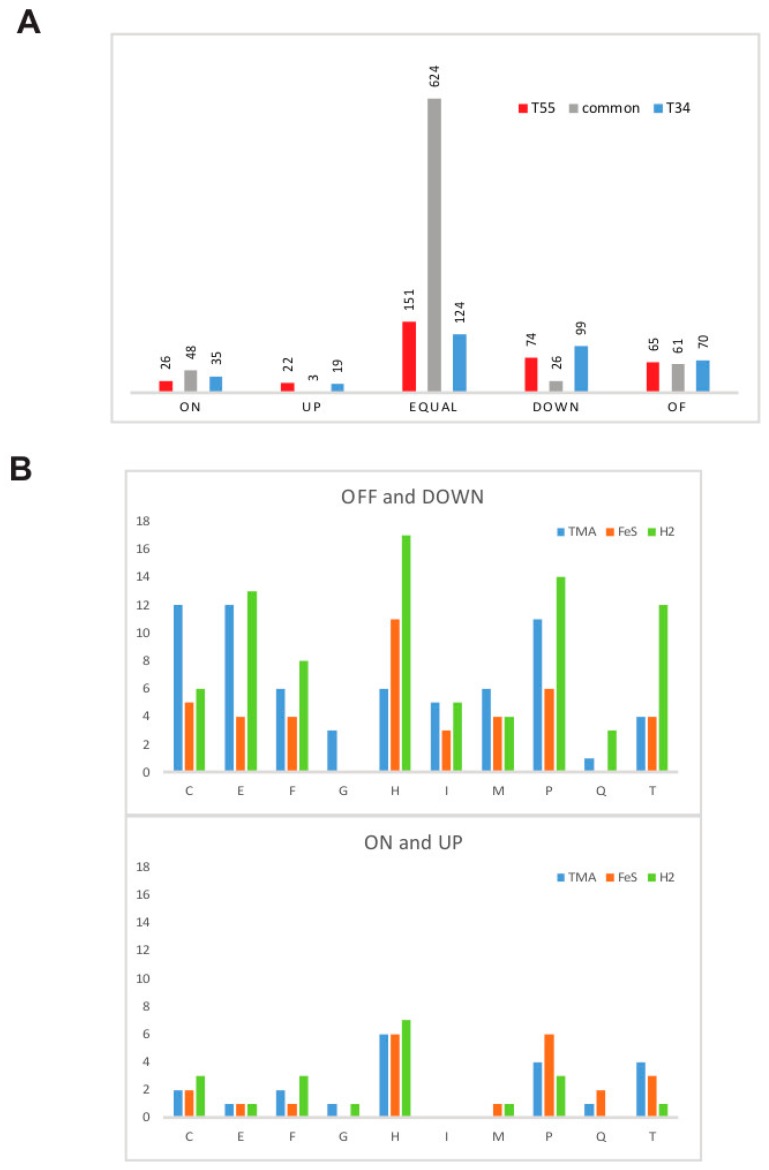
Protein abundance changes in response to non-optimal conditions. (**A**) Comparison of response to supra- and suboptimal temperature conditions. The bars show the number of proteins. (**B**) Comparison of the response to different growth factors for different functional categories. The bars show the number of proteins. Upper panel show upregulated proteins and bottom panel show downregulated proteins. Functional categories are by one letter code as follows: E—Amino acid metabolism; F—nucleotide metabolism; G—Carbohydrate metabolism; H—Coenzyme metabolism; I —Lipid metabolism, M—Cell wall/membrane/envelop biogenesis; P—Inorganic ion transport and metabolism; Q—secondary metabolism.

The PCA described above shows that sub- or supraoptimal temperature contribute jointly and significantly to the variation of the protein abundances, suggesting that many changes in protein abundance in these conditions are correlated. Indeed, 138 proteins are similarly affected by supra- and suboptimal temperatures (Figure 4). This result does not appear surprising given that *M. thermophilum* AMET1 grows equally poorly at 55 °C and 34 °C [3]. This list is dominated by the proteins that are either significantly downregulated or not detectable at all under these conditions (Figure 4, Table 2). Specifically, we observed strong downregulation of proteins involved in pyrrolysine biosynthesis [40], a rare, non-canonical amino acid that is present in two of the most abundant proteins of *M. thermophilum* AMET1 (dimethylamine methyltransferase MtbB AMET1_0722/AMET1_0723 and trimethylamine methyltransferase MttB AMET1_0103/AMET1_0104) involved in methyl group reduction (Table 2). We also detected significant decrease in the abundance of two glycosyltransferases, suggesting that change in temperature affects surface S-layer composition. It has to be taken into account that high or low temperature at extreme medium alkalinity makes a large difference on resistance of the external glycoproteins to hydrolytic damage.

The ribosome properties also could be affected because we observed significant upregulation of ribosomal protein L31E and the converse downregulation of L21E (Table 2). These two ribosomal proteins are encoded in a minor ribosomal superoperon, in which the abundances of the remaining gene products do not change significantly (Table S1). Most of the ribosomal proteins in this superoperon are archaea-specific or shared with eukaryotes but not with bacteria. Both L31E and L21E are assembled relatively late into the eukaryotic large subunit and, unlike the core ribosomal proteins, potentially are the subject to tuning [41]. In eukaryotes L31E is involved in the interaction with ribosome-associated chaperones, which act first to help folding nascent proteins exiting the ribosome [42]; the archaeal orthologue of L31E is likely to perform a similar function, which would account for its heat-induced expression. From the archaeal ribosome structure, it is clear that L21E is one of the 5 ribosomal proteins that stabilize the attachment of 5S rRNA to the large subunit [43]. The role in L21E protein in temperature change response remains unknown.

In both conditions, we observed induction of several proteins from a putative integrated His2-like spindle-shaped halovirus (AMET1_0519-AMET1_0565) (Table 2, Table S1). Induction of integrated elements under various stress conditions is a well-known phenomenon [44,45,46]. Finally, two transcriptional regulators of ArsR family were found to be significantly affected in the opposite directions (Table 2). The aforementioned AMET1_0092, one of the most abundant proteins, is downregulated, whereas AMET1_0853 is upregulated. The AMET1_0092 protein belongs to arCOG01060 and shows a patchy distribution in archaea, although it is present in many methanogens; in contrast, AMET1_0853 belongs to arCOG01684 which is represented in most euryarchaea [47]. Both archaeal heat-responsive regulators characterized to date, Phr from *Pyrococcus furiosus* [48] and HSR1 from *Archaeoglobus fulgidus* [49], belong to arCOG01684. These transcriptional regulators have been shown to substantially induce the expression of the HSP20 family chaperone and CDC48, the AAA ATPase involved in protein folding and degradation control [48,49]. HSR1 is upregulated in heat stress in *A. fulgidus* [49] and AMET1_0853 is also upregulated much more in T55 conditions (Table 2). Thus, the proteomic data provide additional evidence of the involvement of these regulators in heat stress response in a distinct class of archaea. These findings are consistent with the previous reconstruction of the LACA gene set, which includes the key components of this response, namely, HSR1 family of arCOG01684, CDC48 ATPase of arCOG01308 and thermosome subunits of arCOG01257 [50] and with the observation that these protein families evolve largely at the expected evolutionary rates and are minimally prone to horizontal gene transfer [14]. Taken together, these lines of evidence suggest that at least some of the mechanisms for the regulation of heat shock protein expression are ancestral in Euryarchaeota and possibly, in all archaea.

Apart from those proteins that show similar responses to both supra- and suboptimal temperatures, many proteins respond specifically to either T55 or T34 conditions (Figure 4, Table 2). The thermosome (HSP60 family chaperonins) heat induction has been demonstrated first in *Pyrodictium occultum* [51] and then in other archaea including *Pyrococcus furiosus* [52], *Halobacterium salinarum* [53] and *Sulfolobus shibatae* [54]. Two predicted thermosome subunits are also strongly and specifically upregulated in *M. thermophilum* AMET1 under T55 (Table 2). In agreement with the demonstrated role of the Phr-HSR1 family transcriptional regulators, we observed significant upregulation of CDC48 ATPase in T55, presumably due to the effect of the transcriptional regulator AMET1_0853. One of the most abundant proteins at T55 is AMET1_1336, which is currently annotated as “hypothetical protein” and can be confidently shown to contain a helix-turn-helix domain (HHpred probability 93%). This protein is below detection level under the optimal conditions but has a nemPAI value of 55 at T55 and 230 in the FeS conditions, where it is the most abundant protein. Orthologues of this protein are currently undetectable in archaea but are readily identifiable in many bacteria, including those of the genus *Staphylococcus* where it encoded in pathogenicity islands. Thus, this protein might play an important role in the regulation of the expression of integrated mobile elements under stress. 

Compared to T55, T34 results in down-regulation of a greater number of proteins involved in house-keeping processes, such as translation, rRNA modification and replication (Table 2, Table S1). Among the few upregulated proteins, there was no readily interpretable trend. Notably, however, AMET1_1156, a member of the HSP20 family of molecular chaperones is significantly upregulated, whereas its paralog AMET1_1255 is significantly downregulated under the T34 conditions. It remains to be determined if the transcriptional regulator AMET1_0853 is responsible for the regulation of the expression of these chaperones. Most bacteria and many mesophilic archaea encode the cold shock protein A (CspA), a RNA-binding protein known to be specifically involved in cold stress response, whereas most thermophiles lack this protein [55]. Both *M. thermophilum* AMET1 and HMET1 do not encode CspA. Recently, however, it has been shown that another RNA-binding protein, which contains a TRAM (named after uridine methylase TRM2 and the MiaB families of tRNA-modifying enzymes) domain, can function as a CspA analogue in thermophilic archaea [56,57]. Indeed, *M. thermophilum* AMET1 encodes two members of this family of TRAM domain proteins (AMET1_0306 and AMET1_1194) and both of them show moderately elevated abundance at T34 (Table S1).

### 3.5. Response to Growth Factor Changes

All three conditions that involved growth factor change, namely TMA, FeS and H_2_, appeared to be suboptimal and even stressful for *M. thermophilum* AMET1, affecting many proteins involved in central metabolic pathways. As with temperature stress, most of the affected proteins are downregulated, with a marginally greater effect in H_2_ conditions compared to the FeS conditions and only few upregulated proteins were identified for each condition (Figure 4B). No differences in the number of upregulated proteins have been observed (Figure 4B).

The use of TMA instead of MeOH as the *e*-acceptor in the medium causes several predictable changes in the methyl-reduction pathways. The methylated-thiol corrinoid protein AMET1_1049 that is likely specific for methanol (MtaC) is downregulated, whereas several methylamine utilization proteins are upregulated, including one that is specific for TMA (MttC) and shows the largest fold change (Table 3). The significantly downregulated transcriptional regulator AMET1_0372 might be responsible for the metabolic adjustment (Table 3). This protein belongs to arCOG01345 (COG03388), which is represented in the majority of *Halobacteria*, *Methanomicrobia* and *Thermoplasmata* but rarely in other groups of euryarchaea. Such conservation in several major archaeal lineages implies involvement of this regulator in some basic house-keeping pathways. 

In both the FeS and H_2_ conditions, key methyl-reduction pathways are negatively affected. Similar to the T55 and T34 conditions, this effect appears to be achieved by the downregulation of pyrrolysine biosynthesis (Table 3). In addition, FeS causes downregulation of both methanogenic corrinoid protein MtaC paralogs AMET1_1049 and AMET1_0748, which are likely involved in methanol reduction. Both proteins, especially AMET1_1049, are abundant at normal conditions. Both FeS and H_2_ conditions, as expected, affect iron uptake and formation of Fe-S clusters, which are upregulated. However, under these two conditions, different ABC transporters are induced (Table 3). In the presence of H_2_, *M. thermophilum* AMET1 is expected to reduce motility because of the significant downregulation of chemotaxis proteins and FlaH protein, the KaiC-like ATPase involved in the archaellum assembly [58]. 

### 3.6. Positive and Negative Correlations between Abundances of Paralogous Proteins

Compared to its closest relatives, aerobic heterotrophic haloarchaea, anaerobic methanogenic *M. thermophilum* AMET1 has a relatively compact genome. Nevertheless, 178 of the 1084 arCOGs represented in this organism have 2 or more paralogs, comprising 639 out of the 1546 proteins. In an attempt to gain insight into the roles of paralogs in the cellular physiology of AMET1, the following analysis was performed. We selected 824 proteins that showed significant variation of their abundance between the 6 experimental conditions and grouped them into paralogous families (according to the arCOG classification) and singletons (Table S3). In this set, 238 proteins formed 75 paralogous families containing proteins that could be distinguished at the proteomics level, whereas the remaining 586 were singletons. For each pair of paralogs within a paralogous family, Pearson correlation coefficient between their coarse-grained abundance profiles was computed. The distribution of the correlation coefficients was compared to the distribution obtained for 10,000 pairs of randomly chosen singletons. This analysis showed that, compared to singletons, the abundance profiles of paralogs were characterized by a marked excess of highly correlated (*r_Pearson_* > 0.8) as well as anti-correlated (*r_Pearson_* < −0.8) pairs of profiles (10.5% and 12.5% vs. the expected 3.2% and 3.8%, respectively). This structure of correlations between the expression profiles of paralogs implies a dual role of gene paralogization in evolution. The positively correlated duplicated genes appear to be co-regulated and accordingly, can be inferred to function synergistically (as iso-enzymes?), providing physiological robustness and/or increased protein dosage. In contrast, the anti-correlated paralogs seem to be counter-regulated and, presumably, perform complementary functions under different physiological conditions.

As a case in point for the apparent synergy between paralogs, we identified strong correlation between the abundances of predicted glycosyltransferases AMET1_0970, AMET1_0957, AMET1_0963, which all belong to arCOG01403, are encoded in the same predicted operon and are, most likely, coregulated given that they are involved in the same pathway of exopolysaccharide biosynthesis. In contrast, another member of the same paralogous family, AMET1_1410, is encoded in a different locus and is anticorrelated with the former three paralogs. Some paralogous proteins can be tightly co-regulated even though the respective genes are separated on the chromosome, for example, AMET1_0699 and AMET1_0151, homologs of periplasmic components of the ABC-type Fe^3+^-hydroxamate transport system. FtsZ family proteins, which are key components of cell division machinery, are known to form two ancestral archaeal clades FtsZ1 and FtsZ2 [59]. These two paralogs are likely subfunctionalized because only one, FtsZ1, appears to be required for cell division under normal conditions in archaea [59]. In agreement to this subfunctionalization, FtsZ1 (AMET1_0742) is highly expressed but anticorrelated with FtsZ2 (AMET1_1349) under different conditions.

## 4. Conclusions

Proteomic analysis of *M. thermophilum* AMET1 confirms that this extremely haloalkaliphilic and moderately thermophilic methanogen deploys a nearly complete repertoire of proteins involved in key methyl-reduction pathways as well as enzymes for biosynthesis of all amino acids, nucleotides and cofactors and transporters for all essential ions. We did not detect enzymes that could comprise any unknown major biosynthetic pathways although several gaps remain in the *M. thermophilum* AMET1 metabolic map. Proteins that are involved in methanol reduction are highly abundant under standard growth conditions (MeOH + formate, 48 °C) but downregulated under sub-optimal conditions. Apparently, the key mechanism of this metabolic switch is downregulation of pyrrolysine biosynthesis genes which are required for translation of methylamine methyltransferase proteins containing this non-canonical amino acid. The high level of UspA family proteins in all tested conditions is compatible with their role in the adaptation to high salt concentrations [3]. Furthermore, we predict that AMET1_0636, one of the most abundant proteins in *M. thermophilum* AMET1 cells in all the conditions, is an RNA chaperone. Proteins with the RNA chaperone functions are poorly characterized in general and remain virtually unknown in archaea. These observations suggest that RNA chaperone activity could be essential under high salt conditions. Considerable parts of the *M. thermophilum* AMET1 genome remain “silent” under optimal condition but are activated under stress, especially heat and cold shock; some of these “silent islands” correspond to integrated mobile genetic elements. 

We identified several key proteins that are upregulated at suboptimal and supraoptimal temperatures and found that they belong to the same protein families that respond to these conditions in other archaea and even bacteria. These proteins include molecular chaperones (heat shock proteins), the AAA ATPase CDC48 and heat shock transcriptional regulator for the T55 conditions and a TRAM domain-containing putative RNA-binding protein for the T34 conditions. These findings are compatible with an ancient origin of the universal heat shock response, perhaps, preceding the Last Universal Cellular Ancestor, whereas the regulation of this response could have evolved as early as the common ancestor of euryarchaea. 

Despite having a relatively compact genome, *M. thermophilum* AMET1 encodes a number of paralogs which show non-randomly correlated or anti-correlated abundances. Thus, proteomic analysis seems to allow identification of paralogs that appear to be co-regulated and function synergistically and those that are differentially regulated and could perform complementary functions.

Surprisingly, the protein abundance data for HMET1 substantially differ from those obtained for all 6 conditions tested for *M. thermophilum* AMET1, suggesting that, even in closely related organisms with the same type of central metabolism that share the same ecological niche (except for the salt composition resulting in a very different osmotic and pH conditions), protein abundance profiles can diverge relatively fast.

The present proteomic analysis sheds light on the environmental adaptation strategies in the recently discovered triple extremophilic methanogens, reveals common mechanisms of response to heat and cold shock in euryarchaea and yields many experimentally testable predictions of protein functions.

## Figures and Tables

**Table 1:** Highly abundant proteins under optimal growth conditions in *Methanonatronarchaeum thermophilum* AMET1.

Genbank Locus ID	Protein Name	Annotation	NemPAI Base Value
**Methanogenesis related proteins**			
AMET1_1459	McrB	Methyl coenzyme M reductase, beta subunit	11.7
AMET1_1461	McrG	Methyl coenzyme M reductase, gamma subunit	68.7 *
AMET1_1049	MtaC	Methanogenic corrinoid protein MtaC	78.6 *
AMET1_0104	MttB1	Trimethylamine-corrinoid methyltransferase	68.4 *
AMET1_0460	MtmB	Monomethylamine methyltransferase	53.6 *
AMET1_0747	MtaB	Methanol-cobalamin methyltransferase B subunit	9.2
AMET1_0264	IlvH	Acetolactate synthase, small subunit	9.0
**Translation related proteins**			
AMET1_0496	RplD	Ribosomal protein L4	10.3
AMET1_0619	RPS28A	Ribosomal protein S28E/S33	18.6
AMET1_0046	RPL20A	Ribosomal protein L20A	10.3
AMET1_0579	TEF1	Translation elongation factor EF-1 alpha, GTPase	9.5
**DNA and RNA binding protein**			
AMET1_0176	LSM1	Small nuclear ribonucleoprotein	13.2
AMET1_0379	Ssh10b	Archaeal DNA-binding protein Alba	161.1 *
AMET1_1312	HHT1	Histones H3 and H4	22.6
**Chaperones**			
AMET1_1255	IbpA	Molecular chaperone, HSP20 family,	9.8
AMET1_1030	-	Nucleotide-binding protein, UspA family	43.2*
**Biological function unknown**			
AMET1_0092	-	Transcriptional regulator, ArsR family	56.8 *
AMET1_0509	-	CBS domain	10.0
AMET1_0343	-	CBS domain	9.0
AMET1_0636	-	Uncharacterized protein	39.0 *

* Proteins that belong to super-abundant heavy tail of the abundance distribution.

**Table 2:** Selected proteins involved in temperature change response in *M. thermophilum* AMET1.

Locus ^#^	Description	Change ^$^	nemPAI or (Fold Change) *
**T55 (maximum growth temperature)**			
AMET1_1336	HTH domain containing protein	ON	55.2
**AMET1_1130**	Thermosome subunit, GroEL/HSP60 family	UP	(15.8)
**AMET1_1197**	Thermosome subunit, GroEL/HSP60 family	UP	(8.5)
**AMET1_0742**	Cell division GTPase FtsZ	UP	(5.8)
**AMET1_0032**	Desulfoferredoxin	UP	(9.7)
**AMET1_0852**	ATPase of the AAA+ class, CDC48 family	UP	(4.0)
**AMET1_0493**	Ribosomal protein S19	DOWN	(11)
**T34 (minimum growth temperature)**			
**AMET1_0938**	DNA replication ATPase HolB, small subunit	DOWN	(19)
**AMET1_1237**	Wybutosine biosynthesis enzyme Trm5	DOWN	(16)
AMET1_1255	Molecular chaperone, HSP20 family	DOWN	(5.3)
**AMET1_0053**	16S rRNA N6-dimethyltransferase RsmA/KsgA/DIM1	OFF	−0.3
AMET1_0781	SAM-dependent methyltransferase	UP	(10.7)
AMET1_0225	Coproporphyrinogen III oxidase HemN	UP	(5.8)
AMET1_0071	Translation elongation factor EF-1 beta	UP	(5.2)
**AMET1_1156**	Molecular chaperone, HSP20 family	ON	0.29
**Both T55 and T34**			
AMET1_0168	(2R,3R)-3-methylornithine synthase PylB	DOWN	(56.5), (5.1)
AMET1_0221	Pyrrolysyl-tRNA-synthetase PylS	DOWN	(36), (6.5)
AMET1_0092	Transcriptional regulator, ArsR family	DOWN	(5.7), (3.2)
AMET1_0050	Ribosomal protein L21E	DOWN	(3.8), (6.2)
**AMET1_0428**	hypothetical protein	OFF	−1.6
**AMET1_0965**	Glycosyltransferase family 1	OFF	−1.4
**AMET1_0970**	Glycosyltransferase family 1	OFF	−1.0
**AMET1_0552**	hypothetical protein	ON	1.11, 0.47
**AMET1_0548**	hypothetical protein	ON	0.71, 0.80
**AMET1_0532**	hypothetical protein	ON	0.89, 0.3
AMET1_0853	Transcriptional regulator, ArsR family	UP	(9.6), (4.2)
AMET1_0044	Ribosomal protein L31E	UP	(3.3), (3.7)

^#^ Proteins that change the abundance in the respective conditions only and not affected in other conditions are highlighted by bold and a larger font. ^$^ “ON”: the protein is not detected under the baseline condition but appears under the alternative condition; “OFF”: the protein is detected under the baseline condition but not under the alternative condition; “UP”: the protein is more abundant under the alternative condition compared to the baseline condition by a factor of more than 3.16; “DOWN”: the protein is less abundant under the alternative condition compared to the baseline condition by a factor of more than 3.16. * The fold change is indicated in parenthesis; comma separates values of change at T55 and T34 conditions; if a protein is “ON”, its nemPAI value for the respective condition is indicated; if a protein is “DOWN”, its nemPAI value at optimal condition is indicated.

**Table 3:** Selected proteins involved in growth factor change response in *M. thermophilum* AMET1.

Locus ^#^	Description	Change ^$^	nemPAI or (Fold Change) *
**TMA**			
AMET1_1049	Methanogenic corrinoid protein MtaC	DOWN	(27.2)
AMET1_0372	Predicted transcriptional regulator	DOWN	(23.2)
**AMET1_0479**	Ribosomal protein L19E	DOWN	(15.4)
**AMET1_1538**	Glutamine synthetase	OFF	−1.2
**AMET1_0199**	Ribosomal protein L37AE/L43A	OFF	−0.4
**AMET1_1486**	Multisubunit Na+/H+ antiporter, MnhG subunit	OFF	−0.26
**AMET1_0038**	ACT domain-containing protein	ON	0.18
**AMET1_1329**	Uncharacterized protein	ON	0.18
**AMET1_0105**	Trimethylamine corrinoid protein MtbC1	UP	(7.1)
AMET1_0722	Dimethylamine methyltransferase MtbB	UP	(4.9)
**AMET1_0460**	Monomethylamine methyltransferase MtmB	UP	(4.4)
**FeS**			
AMET1_1049	Methanogenic corrinoid protein MtaC	DOWN	(11.8)
AMET1_0222	(2R,3R)-3-methylornithine synthase PylB	DOWN	(10/8)
**AMET1_0748**	Methanogenic corrinoid protein MtaC	DOWN	(7.0)
AMET1_0462	Fe-S cluster biogenesis protein NfuA, 4Fe-4S-binding domain	UP	(13.7)
AMET1_1183	Energy-coupling factor transporter ATP-binding protein EcfA2	UP	(5.5)
AMET1_0093	ABC-type cobalamin/Fe3+-siderophores transport system, ATPase component	UP	(5.4)
AMET1_1336	HTH domain containing protein	ON	230.9
AMET1_0698	ABC-type Fe3+-hydroxamate transport system, periplasmic component	ON	1.0
**H_2_**			
**AMET1_0168**	(2R,3R)-3-methylornithine synthase PylB	OFF	−1.3
AMET1_0222	(2R,3R)-3-methylornithine synthase PylB	OFF	−0.4
**AMET1_1113**	Methylase of chemotaxis methyl-accepting protein	OFF	−0.4
AMET1_0575	Chemotaxis signal transduction protein CheW	OFF	−0.3
**AMET1_0021**	Chemotaxis signal transduction protein CheW	OFF	−0.2
**AMET1_1117**	Chemotaxis protein histidine kinase CheA	DOWN	(30.7)
**AMET1_0325**	Chromosome partition protein Smc	DOWN	(28.0)
**AMET1_1115**	Chemotaxis protein CheC, flagellar motor switch protein	DOWN	(13.1)
**AMET1_0923**	KaiC family ATPase FlaH involved in archaellum biogenesis	DOWN	(8.5)
AMET1_0181	Nucleoside 2-deoxyribosyltransferase	UP	(6.6)
AMET1_0462	Fe-S cluster biogenesis protein NfuA, 4Fe-4S-binding domain	UP	(6.2)
**AMET1_0880**	ABC-type metal ion transport system, periplasmic component/surface adhesin LraI	UP	(5.9)
**AMET1_0879**	ABC-type Mn/Zn transport system, ATPase component ZhuC	UP	(4.8)

^#^ Proteins that change the abundance in the respective conditions only and not affected in other conditions are highlighted by bold and a larger font. ^$^ “ON”: the protein is not detected under the baseline condition but appears under the alternative condition; “OFF”: the protein is detected under the baseline condition but not under the alternative condition; “UP”: the protein is more abundant under the alternative condition compared to the baseline condition by a factor of more than 3.16; “DOWN”: the protein is less abundant under the alternative condition compared to the baseline condition by a factor of more than 3.16. * The fold change is indicated in parenthesis; if a protein is “O”, its nemPAI value for the respective condition is indicated; if a protein is “DOWN”, its nemPAI value at optimal condition is indicated.

## Supplementary Materials

The following are available online at http://www.mdpi.com/2073-4425/9/2/28/s1, Table S1: Protein abundances (nemPAI) mapped to *M. thermophilum* AMET1 genome and their change in different experimental conditions. Table S2: List of homologs for the putative RNA chaperon. Table S3: Comparison of paralog abundance profiles. Figure S1: Distribution of relative protein abundances (nemPAI) values. Figure S2: UbiD co-localization with predicted archaeal aconitase AcoX.

## References

[1] Offre P., Spang A., Schleper C. (2013). Archaea in biogeochemical cycles. Annu. Rev. Microbiol..

[2] Adam P.S., Borrel G., Brochier-Armanet C., Gribaldo S. (2017). The growing tree of archaea: New perspectives on their diversity, evolution and ecology. ISME J..

[3] Sorokin D.Y., Makarova K.S., Abbas B., Ferrer M., Golyshin P.N., Galinski E.A., Ciordia S., Mena M.C., Merkel A.Y., Wolf Y.I. (2017). Discovery of extremely halophilic, methyl-reducing euryarchaea provides insights into the evolutionary origin of methanogenesis. Nat. Microbiol..

[4] Borrel G., O’Toole P.W., Harris H.M., Peyret P., Brugere J.F., Gribaldo S. (2013). Phylogenomic data support a seventh order of methylotrophic methanogens and provide insights into the evolution of methanogenesis. Genome Biol. Evol..

[5] Borrel G., Adam P.S., Gribaldo S. (2016). Methanogenesis and the wood-ljungdahl pathway: An ancient, versatile and fragile association. Genome Biol. Evol..

[6] Ferrer M., Werner J., Chernikova T.N., Bargiela R., Fernandez L., La Cono V., Waldmann J., Teeling H., Golyshina O.V., Glockner F.O. (2012). Unveiling microbial life in the new deep-sea hypersaline lake Thetis. Part ii: A metagenomic study. Environ. Microbiol..

[7] Jiang H., Dong H., Yu B., Liu X., Li Y., Ji S., Zhang C.L. (2007). Microbial response to salinity change in lake Chaka, a hypersaline lake on tibetan plateau. Environ. Microbiol..

[8] Eder W., Schmidt M., Koch M., Garbe-Schonberg D., Huber R. (2002). Prokaryotic phylogenetic diversity and corresponding geochemical data of the brine-seawater interface of the Shaban Deep, Red Sea. Environ. Microbiol..

[9] Sorokin D.Y., Abbas B., Geleijnse M., Pimenov N.V., Sukhacheva M.V., van Loosdrecht M.C. (2015). Methanogenesis at extremely haloalkaline conditions in the soda lakes of Kulunda steppe (Altai, Russia). FEMS Microbiol. Ecol..

[10] Sorokin D.Y., Abbas B., Merkel A.Y., Rijpstra W.I., Damste J.S., Sukhacheva M.V., van Loosdrecht M.C. (2015). *Methanosalsum natronophilum* sp. nov. and *Methanocalculus alkaliphilus* sp. nov., haloalkaliphilic methanogens from hypersaline soda lakes. Int. J. Syst. Evol. Microbiol..

[11] Torgerson W.S. (1958). Theory and Methods of Scaling.

[12] Arike L., Peil L. (2014). Spectral counting label-free proteomics. Methods Mol. Biol..

[13] Team R.C. (2013). R: A Language and Environment for Statistical Computing.

[14] Petitjean C., Makarova K.S., Wolf Y.I., Koonin E.V. (2017). Extreme deviations from expected evolutionary rates in archaeal protein families. Genome Biol. Evol..

[15] Altschul S.F., Madden T.L., Schaffer A.A., Zhang J., Zhang Z., Miller W., Lipman D.J. (1997). Gapped blast and psi-blast: A new generation of protein database search programs. Nucleic Acids Res..

[16] Edgar R.C. (2004). Muscle: Multiple sequence alignment with high accuracy and high throughput. Nucleic Acids Res..

[17] Drozdetskiy A., Cole C., Procter J., Barton G.J. (2015). Jpred4: A protein secondary structure prediction server. Nucleic Acids Res..

[18] Krogh A., Larsson B., von Heijne G., Sonnhammer E.L. (2001). Predicting transmembrane protein topology with a Hidden Markov Model: Application to complete genomes. J. Mol. Biol..

[19] Bath C., Cukalac T., Porter K., Dyall-Smith M.L. (2006). His1 and His2 are distantly related, spindle-shaped haloviruses belonging to the novel virus group, salterprovirus. Virology.

[20] Laurens N., Driessen R.P., Heller I., Vorselen D., Noom M.C., Hol F.J., White M.F., Dame R.T., Wuite G.J. (2012). Alba shapes the archaeal genome using a delicate balance of bridging and stiffening the DNA. Nat. Commun..

[21] Friedrich M.W. (2005). Methyl-coenzyme m reductase genes: Unique functional markers for methanogenic and anaerobic methane-oxidizing archaea. Methods Enzymol..

[22] Shima S., Krueger M., Weinert T., Demmer U., Kahnt J., Thauer R.K., Ermler U. (2011). Structure of a methyl-coenzyme m reductase from black sea mats that oxidize methane anaerobically. Nature.

[23] Enoki M., Shinzato N., Sato H., Nakamura K., Kamagata Y. (2011). Comparative proteomic analysis of *Methanothermobacter themautotrophicus* Δh in pure culture and in co-culture with a butyrate-oxidizing bacterium. PLoS ONE.

[24] Williams T.J., Burg D.W., Ertan H., Raftery M.J., Poljak A., Guilhaus M., Cavicchioli R. (2010). Global proteomic analysis of the insoluble, soluble and supernatant fractions of the psychrophilic archaeon *Methanococcoides burtonii*. Part ii: The effect of different methylated growth substrates. J. Proteome Res..

[25] Xia Q., Hendrickson E.L., Zhang Y., Wang T., Taub F., Moore B.C., Porat I., Whitman W.B., Hackett M., Leigh J.A. (2006). Quantitative proteomics of the archaeon *Methanococcus maripaludis* validated by microarray analysis and real time pcr. Mol. Cell. Proteom..

[26] Ereno-Orbea J., Oyenarte I., Martinez-Cruz L.A. (2013). CBS domains: Ligand binding sites and conformational variability. Arch. Biochem. Biophys..

[27] Doetsch M., Schroeder R., Furtig B. (2011). Transient RNA-protein interactions in RNA folding. FEBS J..

[28] Schey K.L., Grey A.C., Nicklay J.J. (2013). Mass spectrometry of membrane proteins: A focus on aquaporins. Biochemistry.

[29] White R.H. (2004). L-aspartate semialdehyde and a 6-deoxy-5-ketohexose 1-phosphate are the precursors to the aromatic amino acids in *Methanocaldococcus jannaschii*. Biochemistry.

[30] Marshall S.A., Payne K.A.P., Leys D. (2017). The UbiX-UbiD system: The biosynthesis and use of prenylated flavin (prfmn). Arch. Biochem. Biophys..

[31] White M.D., Payne K.A., Fisher K., Marshall S.A., Parker D., Rattray N.J., Trivedi D.K., Goodacre R., Rigby S.E., Scrutton N.S. (2015). UbiX is a flavin prenyltransferase required for bacterial ubiquinone biosynthesis. Nature.

[32] Makarova K.S., Koonin E.V. (2003). Filling a gap in the central metabolism of archaea: Prediction of a novel aconitase by comparative-genomic analysis. FEMS Microbiol. Lett..

[33] Elling F.J., Becker K.W., Konneke M., Schroder J.M., Kellermann M.Y., Thomm M., Hinrichs K.U. (2016). Respiratory quinones in archaea: Phylogenetic distribution and application as biomarkers in the marine environment. Environ. Microbiol..

[34] Tietze M., Beuchle A., Lamla I., Orth N., Dehler M., Greiner G., Beifuss U. (2003). Redox potentials of methanophenazine and CoB-S-S-CoM, factors involved in electron transport in methanogenic archaea. Chembiochem.

[35] Cavicchioli R., Thomas T., Curmi P.M. (2000). Cold stress response in archaea. Extremophiles.

[36] Bowman J.B. (2008). Genomic analysis of psychrophilic prokaryotes. Psychrophiles: From Biodiversity to Biotechnology.

[37] Richter K., Haslbeck M., Buchner J. (2010). The heat shock response: Life on the verge of death. Mol. Cell.

[38] Macario A.J., Conway de Macario E. (2007). Molecular chaperones: Multiple functions, pathologies and potential applications. Front. Biosci. J. Virtual Libr..

[39] Williams T.J., Liao Y., Ye J., Kuchel R.P., Poljak A., Raftery M.J., Cavicchioli R. (2017). Cold adaptation of the antarctic haloarchaea *Halohasta litchfieldiae* and *Halorubrum lacusprofundi*. Environ. Microbiol..

[40] Gaston M.A., Jiang R., Krzycki J.A. (2011). Functional context, biosynthesis and genetic encoding of pyrrolysine. Curr. Opin. Microbiol..

[41] Fromont-Racine M., Senger B., Saveanu C., Fasiolo F. (2003). Ribosome assembly in eukaryotes. Gene.

[42] Leidig C., Bange G., Kopp J., Amlacher S., Aravind A., Wickles S., Witte G., Hurt E., Beckmann R., Sinning I. (2013). Structural characterization of a eukaryotic chaperone--the ribosome-associated complex. Nat. Struct. Mol. Biol..

[43] Ban N., Nissen P., Hansen J., Moore P.B., Steitz T.A. (2000). The complete atomic structure of the large ribosomal subunit at 2.4 a resolution. Science.

[44] Howard-Varona C., Hargreaves K.R., Abedon S.T., Sullivan M.B. (2017). Lysogeny in nature: Mechanisms, impact and ecology of temperate phages. ISME J..

[45] Mochizuki T., Sako Y., Prangishvili D. (2011). Provirus induction in hyperthermophilic archaea: Characterization of *Aeropyrum pernix* spindle-shaped virus 1 and *Aeropyrum pernix* ovoid virus 1. J. Bacteriol..

[46] Ratner V.A., Vasil’eva L.A. (1992). The role of mobile genetic elements (MGE) in microevolution. Genetika.

[47] Makarova K.S., Wolf Y.I., Koonin E.V. (2015). Archaeal clusters of orthologous genes (arCOGs): An update and application for analysis of shared features between Thermococcales, Methanococcales and Methanobacteriales. Life.

[48] Vierke G., Engelmann A., Hebbeln C., Thomm M. (2003). A novel archaeal transcriptional regulator of heat shock response. J. Biol. Chem..

[49] Rohlin L., Trent J.D., Salmon K., Kim U., Gunsalus R.P., Liao J.C. (2005). Heat shock response of *Archaeoglobus fulgidus*. J. Bacteriol..

[50] Wolf Y.I., Makarova K.S., Yutin N., Koonin E.V. (2012). Updated clusters of orthologous genes for archaea: A complex ancestor of the archaea and the byways of horizontal gene transfer. Biol. Direct.

[51] Phipps B.M., Hoffmann A., Stetter K.O., Baumeister W. (1991). A novel ATPase complex selectively accumulated upon heat shock is a major cellular component of thermophilic archaebacteria. EMBO J..

[52] Shockley K.R., Ward D.E., Chhabra S.R., Conners S.B., Montero C.I., Kelly R.M. (2003). Heat shock response by the hyperthermophilic archaeon *Pyrococcus furiosus*. Appl. Environ. Microbiol..

[53] Weng R.R., Shu H.W., Chin S.W., Kao Y., Chen T.W., Liao C.C., Tsay Y.G., Ng W.V. (2014). Omics in ecology: Systems level analyses of *Halobacterium salinarum* reveal large-scale temperature-mediated changes and a requirement of CctA for thermotolerance. Omics J. Integr. Biol..

[54] Kagawa H.K., Yaoi T., Brocchieri L., McMillan R.A., Alton T., Trent J.D. (2003). The composition, structure and stability of a group ii chaperonin are temperature regulated in a hyperthermophilic archaeon. Mol. Microbiol..

[55] Giaquinto L., Curmi P.M., Siddiqui K.S., Poljak A., DeLong E., DasSarma S., Cavicchioli R. (2007). Structure and function of cold shock proteins in archaea. J. Bacteriol..

[56] Zhang B., Yue L., Zhou L., Qi L., Li J., Dong X. (2017). Conserved TRAM domain functions as an archaeal cold shock protein via DNA chaperone activity. Front. Microbiol..

[57] Taha, Siddiqui K.S., Campanaro S., Najnin T., Deshpande N., Williams T.J., Aldrich-Wright J., Wilkins M., Curmi P.M., Cavicchioli R. (2016). Single TRAM domain RNA-binding proteins in archaea: Functional insight from Ctr3 from the antarctic methanogen *Methanococcoides burtonii*. Environ. Microbiol..

[58] Chaudhury P., Neiner T., D’Imprima E., Banerjee A., Reindl S., Ghosh A., Arvai A.S., Mills D.J., van der Does C., Tainer J.A. (2016). The nucleotide-dependent interaction of FlaH and FlaI is essential for assembly and function of the archaellum motor. Mol. Microbiol..

[59] Aylett C.H.S., Duggin I.G., Löwe J., Amos L. (2017). The tubulin superfamily in archaea. Prokaryotic Cytoskeletons.

