# Supplementary material for: Proteomic Analysis of Methanonatronarchaeum thermophilum AMET1, a Representative of a Putative New Class of Euryarchaeota, “Methanonatronarchaeia”
Source: Genes (Basel). 2018 Jan 23;9(2):28. doi: 10.3390/genes9020028 (PMC5852551; doi:10.3390/genes9020028)
Supplement: Supplementary file 1 [file genes-09-00028-s001.zip › Figure_S2.pdf]

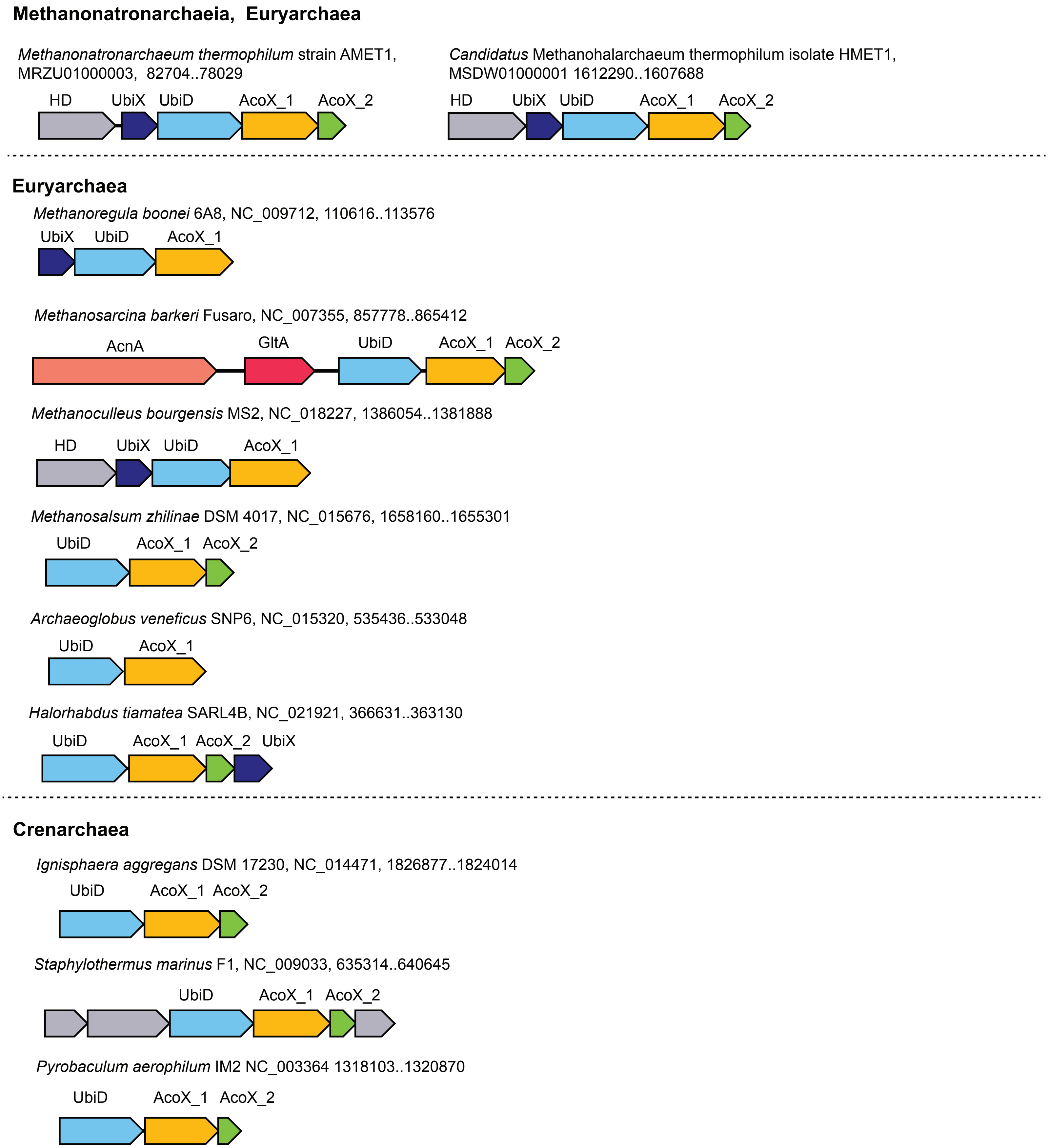

Figure S1: UbiD co-localization with predicted archaeal aconitase AcoX.
For each locus, species name, genome accession number and the respective nucleotide coordinates are indicated.
The genes are shown by arrows. The scale of an arrow is roughly proportional to gene length.
The arrow indicates direction of the respective gene. Homologous genes and domains are color-coded.
Abbreviations and gene names: UbiX and UbiD – decarboxylase system components;
AcoX_1 – predicted aconitase, enzymatic subunit; AcoX_2 – AcoX_1 associated swivelling domain;
AcoA – aconitase A; GltA – citrate synthase; HD – HD family hydrolase.
