# Supplementary material for: Proteomic Analysis of Methanonatronarchaeum thermophilum AMET1, a Representative of a Putative New Class of Euryarchaeota, “Methanonatronarchaeia”
Source: Genes (Basel). 2018 Jan 23;9(2):28. doi: 10.3390/genes9020028 (PMC5852551; doi:10.3390/genes9020028)
Supplement: Supplementary file 1 [file genes-09-00028-s001.zip › Figure_S1.pdf]

A

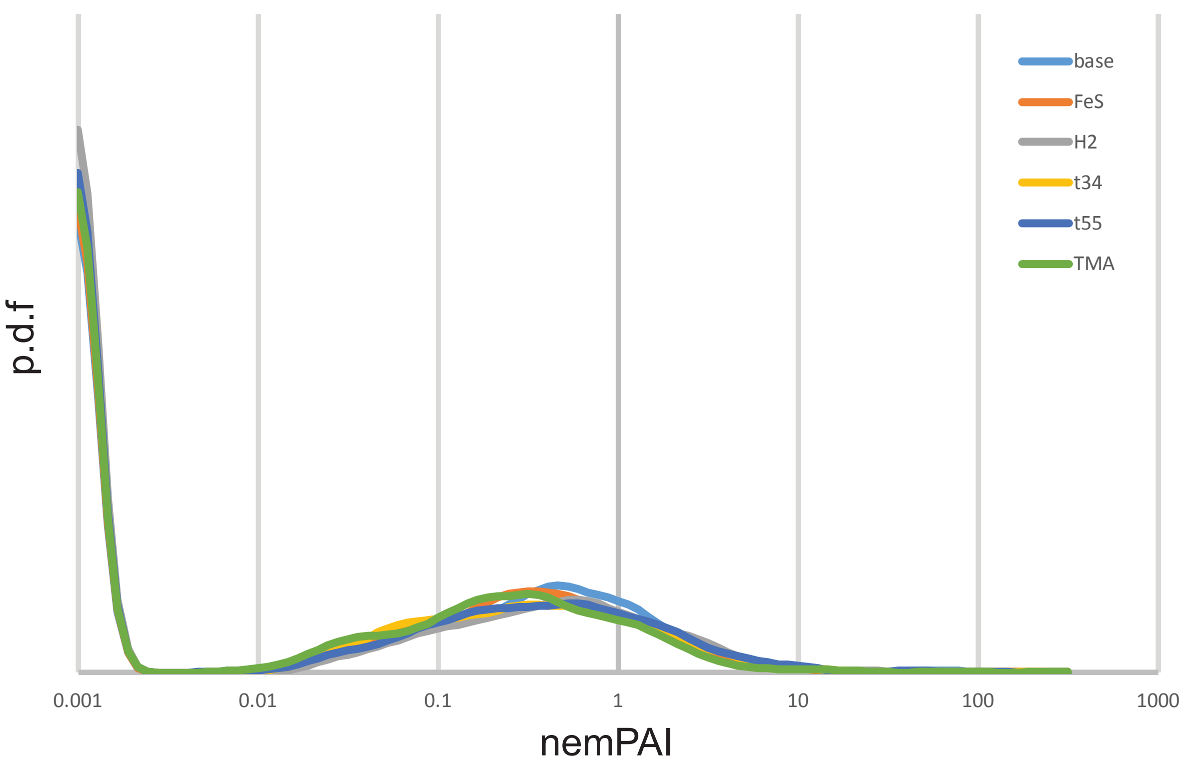

B

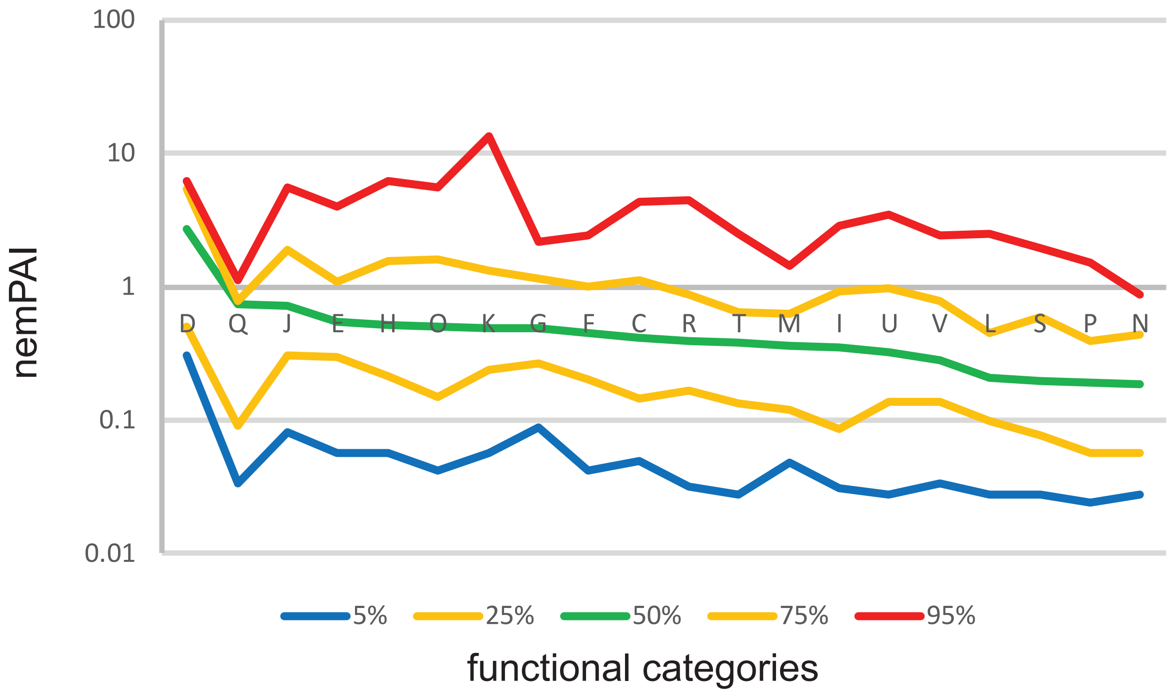

Figure S1: Distribution of relative protein abundances (nemPAI) values

A. Distribution of nemPAI values for all six conditions tested for M. thermophilum AMET1.
Proteins not detected with LC-MS/MS are assigned an arbitrary low nemPAI value of 0.001.

B. Distribution of M. thermophilum AMET1 nemPAI values at optimal growth conditions across functional categories.
The lines indicate (bottom to top) the 5th, 35th, 50th (the median), 75th and 95th percentiles of the nemPAI value distributions.
